# Rosai–Dorfman–Destombes disease of the nervous system: a systematic literature review

**DOI:** 10.1186/s13023-022-02220-0

**Published:** 2022-03-02

**Authors:** Ruham Alshiekh Nasany, Anne S. Reiner, Jasmine H. Francis, Oussama Abla, Katherine S. Panageas, Eli L. Diamond

**Affiliations:** 1 Department of Neurology, Memorial Sloan Kettering Cancer Center, 160 East 53rd Street, 2nd Floor, New York, NY 10022 USA; 2 Department of Epidemiology and Biostatistics, Memorial Sloan Kettering Cancer Center, New York, NY USA; 3 Ophthalmic Oncology Service, Department of Surgery, Memorial Sloan Kettering Cancer Center, New York, NY USA; 4 Division of Hematology/Oncology, The Hospital for Sick Children, Toronto, ON Canada

**Keywords:** Rosai–Dorfman disease, Histiocytosis, MAPK pathway, Systematic review

## Abstract

**Background:**

Rosai–Dorfman–Destombes disease (RDD) is a rare histiocytic disorder with heterogeneous clinical manifestations and rare neurologic involvement. The existing clinical literature about neurologic RDD has yet to be critically examined.

**Methods:**

We performed a four-database English-language systematic literature search for cases of RDD neurohistiocytosis, excluding secondary literature. Individual patient data for neurologic symptoms, disease sites, treatments, and responses were captured. Responses to first-line and second-line surgical interventions, post-surgical radiotherapy, and systemic therapies were analyzed.

**Results:**

Among 4769 articles yielded by literature search, 154 articles were fully reviewed, containing data on 224 patients with neurologic RDD. 128 (83.1%) articles were single case reports. 149 (66.5%) patients were male, 74 (33.5%) female, with a median age of 37.6 years (range 2–79). Presenting neurologic symptoms included headache (45.1%), focal neurological deficits (32.6%), visual symptoms (32.1%), and seizures (24.6%). RDD involvement was multifocal in 32 (14.3%) cases. First-line treatment involved resection in 200 (89.6%) patients, with subsequent progression in 52 (26%), including 41 (78.8%) with unifocal disease. No difference was observed in progression-free survival comparing post-operative radiotherapy to no radiotherapy following partial resection. Chemotherapy given alone as first-line treatment led to complete or partial response in 3/7(43%) patients. Second-line treatments led to complete or partial response in 18/37(37.5%) patients. Mutational data were reported on 10 patients (4.46%).

**Conclusions:**

This review highlights the limited published data about neurologic RDD, which presents with varied symptomatology and outcome. Further study is needed about its mutational landscape, and more effective therapies are needed for recurrent and refractory disease.

**Supplementary Information:**

The online version contains supplementary material available at 10.1186/s13023-022-02220-0.

## Introduction

Rosai–Dorfman–Destombes disease (RDD) is a rare non-Langerhans cell histiocytosis (LCH) that was first described in 1965 by a French pathologist, Pierre Paul Louis Lucien Destombes [1]. RDD was subsequently characterized as a clinic entity with two publications by Drs. Juan Rosai and Ronald Dorfman analyzing four and 34 patients in 1969 and 1972, respectively [2, 3]. Almost half a century later, approximately 1000 RDD cases have been reported in English publications, including an international registry of 423 cases, published in 1990, which remains the largest source of clinical information of RDD [4]. The hallmark finding of RDD upon examination of biopsy material is lesional histiocytes that are immunophenotyped-positive for CD68+ and S100, negative for CD1a, and that demonstrate variable frequency of intracytoplastic trafficking of lymphocytes, or emperipolesis [5].

The etiology of RDD is complex and multifaceted, and there are forms of disease that are familial, associated with autoimmunity or cancer, and occur in isolation [6]. Recently, somatic mutations in the mitogen-activated protein kinase (MAPK) pathway have been found in lesional tissue in a subset of RDD cases, suggesting that at least some forms of RDD are neoplastic [7]. Clinically, RDD is a heterogeneous entity with a wide range of phenotypes, from limited and self-resolving to life-threatening forms. The disease classically presents with bilateral cervical lymphadenopathy, but recently published two case series presenting adult patients with RDD reported extra nodal disease in 92% and 76.5% of their 64 and 32 patients respectively [29, 30]. Neurologic involvement in RDD is rare, estimated in one series to occur in 5% of cases, and is described to involve the calvarium, dura, orbit and facial sinuses, brain parenchyma, and spine [8]. There has been a review of the existing case literature of neurologic RDD with respect to disease localization [9]. There is an absence, however, of collectively analyzed data regarding the spectrum of neurologic RDD manifestations, including clinical symptomatology and specific first- and later-line treatment outcomes. Recent consensus recommendations for the management of RDD cite examples from the literature of treatment strategies and outcomes with selected instances of neurologic RDD but do not critically evaluate the evidence base for managing this entity [6]. Our objective was to characterize and synthesize the existing published English clinical literature about neurologic involvement of RDD including presenting symptoms, sites of neurologic involvement, type and response to treatment. We further sought to elucidate opportunities for learning and investigation about this rare neuro-oncologic disease.

## Methods

### Literature search and selection of included articles

**Fig. 1:**
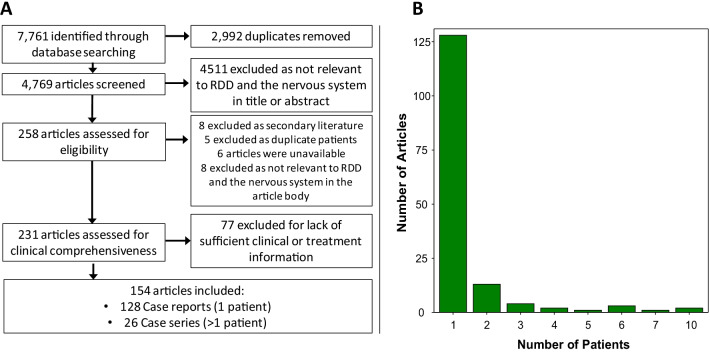
(**A**) Systematic library search, article screening, inclusion and exclusion. (**B**) Histogram demonstrating the number of articles reviewed examining a given number of patients

We conducted systematic review searches (Fig. 1a) in Medline (PubMed), Embase.com, Web of Science (Clarivate Analytics), and the Cochrane Library (Wiley). No date limitations were applied to the search. We searched for articles written in English, excluding conference proceedings and abstracts. For search terms (using headings and keywords), we combined two key concepts using the AND operator: histiocytic diseases (e.g., histiocytosis, Rosai–Dorfman) and neurologic involvement (e.g., central nervous system, brain, spinal cord). See Additional file 2 for the complete list of search terms used. In Medline and Embase, we used the Cochrane filter for excluding animal-only studies [10]. Search results were combined in a bibliographic management tool (EndNote) and duplicates were eliminated following the Bramer Method [11].

### Review of search results and selection of included articles:

The titles and abstracts of the articles identified by the above search were screened by title and abstract for relevance to both RDD and the nervous system. The nervous system was defined as the brain parenchyma, meninges, calvarium, orbit, and spine (including the spinal cord, spinal canal, and spinal meninges). Articles thought to be relevant were then reviewed in their full-text and further screened for inclusion in this study by the following criteria: articles included: (1) were original articles that were relevant to RDD and the nervous system and (2) presented all of the following clinical data: presenting symptoms, sites of disease involvement, treatment, and radiographic response. Secondary literature was excluded.

### Demographic and clinical data

For all included articles, the number of patients reported was documented as well as patient level data including age and sex. Presenting symptoms were categorized as headache, focal neurological deficits, visual symptoms, seizures, cranial neuropathies, cerebellar dysfunction, and cognitive decline, and others. Sites of disease were categorized by involvement of dura, spine, brain parenchyma, orbit, calvarium, or multiple sites. Molecular sequencing data was captured when reported.

### RDD treatment and response data

Methods of treatment were categorized into surgical resection, radiotherapy, steroids, and systemic chemotherapy. When two treatment modalities were implemented as a pre-determined combination (e.g., the decision for surgical resection followed by radiotherapy), this combination was considered a single therapy. By contrast, when a second treatment was implemented as a result of the outcome of the first treatment (e.g., surgical resection and then a decision to implement radiotherapy after recurrence), then these were considered two distinct lines of therapy.

For cases involving surgical resection, the extent of resection was categorized into complete resection, partial resection, or unknown extent of resection. Accordingly, the radiographic response to resection reflected the extent of resection; gross total resection was considered a complete response (CR) and partial resection was considered a partial response (PR). For non-surgical treatments, the best radiographic response to each line of therapy was categorized into (1) complete response (CR) in the cases of documented “complete resolution” of the lesions involving the nervous system, (2) partial response (PR) in the cases where the articles used the term “partial resolution” or when MRI images provided in the included articles demonstrated partial lesional regression, (3) progression of disease (POD) in the cases where articles used the term “progression”, the cases when MRI images provided in the included articles demonstrated lesional growth, or in cases of death, and (4) stable disease (SD) in the cases that did not meet the criteria for PR or POD or where the author of included articles used the term “stable disease”.

### Statistical analysis

The frequencies of implemented first-line treatments were compared across sites of disease. Responses to treatment were summarized by line of treatment for all aggregated disease sites and by site of disease. The frequency of subsequent progression or recurrence (to which we will refer as “progression”) following treatment was also summarized for each line of treatment, treatment category, and site of disease. The Kaplan–Meier method was used to describe progression-free survival (PFS). Follow-up was calculated from date of RDD diagnosis until progression or last follow-up. The log-rank test was used to compare PFS between first-line surgical treatments with and without radiation therapy. Adult and pediatric patients were compared across categorical variables of interest using the chi-squared or Fisher’s test, where appropriate, and were compared across continuous variables of interest using t-tests. Tests were two-sided with a level of statistical significance < 0.05. Analyses were performed in SAS v9.4 (The SAS Institute, Cary, NC) and R v3.6.0 (The R Foundation for Statistical Computing).

## Results

### Article and patient characteristics

The initial literature search yielded 4769 articles, of which 4511 were excluded for lack of relevance to the nervous system or RDD by title and abstract (Fig. 1a). 258 articles were reviewed in full text, and of these 154 articles were included in our final analysis (Fig. 1b). See Additional file 1 for the list of included articles. Of these, 128 (83.1%) were single-case reports presenting one patient each. The remaining articles 26 (16.9%) were case-series presenting 2 or more patients each. A total of 224 patients were analyzed with a mean age of 37.6 years (range 2–79; IQR 22–52.5); 42 (18.75%) were pediatric (< 18 years) and 182 (81.25%) were adult. There were 149 male patients (66.5%) and 74 (33.5%) were female.

### Presenting symptoms and sites of involvement

The most frequent presenting symptoms (Table 1) were headache in 101 cases (45.1%), focal neurological deficits (32.6%), visual symptoms (32.1%), seizures (24.6%), cranial neuropathies (11.6%), cerebellar dysfunction (11.2%), and cognitive decline (5.8%). Nasal fullness, neuro-endocrine dysfunction, and palpable masses were less frequent presentations (5.4% combined). Presenting neurologic symptoms were similar between the adult and pediatric groups, however, occurrence of seizure as the presenting symptoms was significantly more prevalent in adults than in children (27.5% versus 11.9%; *p* = 0.03) while visual and cerebellar symptoms were more prevalent in children than adults (42.9% versus 29.7% and 19% versus 9.3% respectively; both *p* = 0.10). RDD lesions were located in the dura in 111 cases (49.6%), spine in 37 (16.5%), brain parenchyma in 21 (9.4%), orbit in 17 (7.6%), calvarium in 6 (2.7%), and in multiple sites in 32 (14.3%). Sites of disease were significantly different between adults and children (*p* = 0.0001), adults had greater involvement of dura (55.5% versus 23.8%) whereas children had greater involvement of the orbit (23.8% versus 3.9%), brain parenchyma (11.9% versus 8.8%), and spine (21.4% versus 15.4%). Mutational data were reported on 10 patients (4.46%); SLC29A3 was sequenced in 8 of these patients and reported to be wild-type in all cases [12]. Targeted exon sequencing was performed upon BRAF for two patients and in one patient an exon 12 deletion was observed [13, 14].

**Table 1:** Characteristics of patients with neurologic Rosai–Dorfman–Destombes disease (N = 224)

Characteristic	All Patients		Adult		Pediatric		*p* value
	N	%	N	%	N	%	
Age (years)	37.6	2–79	43.8	18–79	11	2–17	< 0.0001
Sex							
Male	149	66.5	127	69.8	22	52.4	0.03
Female	75	33.5	55	30.2	20	47.6	
Presenting symptom(s)							
Headache	101	45.1	85	46.7	16	38.1	0.31
Seizure	55	24.6	50	27.5	5	11.9	0.03
Focal finding	73	32.6	62	34.1	11	26.2	0.33
Cranial nerve deficit	26	11.6	19	10.4	7	16.7	0.29
Ocular/visual symptoms	72	32.1	54	29.7	18	42.9	0.10
Cerebellar symptoms	25	11.2	17	9.3	8	19	0.10
Cognitive decline/dementia	13	5.8	10	5.5	3	7.1	0.71
Other symptoms							
Sinus/nasal symptoms	4	1.8	4	2.2	0	0	1.00
Superficial mass	3	1.3	2	1.1	1	2.4	0.47
Hormonal abnormalities	4	1.8	1	0.5	3	7.1	0.02
Lymphadenopathy	1	0.5	1	0.5	0	0	1.00
Site(s)							
Calvarium	6	2.7	5	2.8	1	2.4	1.00
Dura	111	49.6	101	55.5	10	23.8	0.0002
Spine: dural cord	37	16.5	28	15.4	9	21.4	0.34
Parenchyma	21	9.4	16	8.8	5	11.9	0.56
Orbit	17	7.6	7	3.9	10	23.8	0.0001
Multiple sites	32	14.3	25	13.7	7	16.7	0.62

^*^p value comparing variables across adult and pediatric populations. T-test for age and Chisquared or Fisher's test as appropriate for other variables

### First-line treatments and responses

**Fig. 2:**
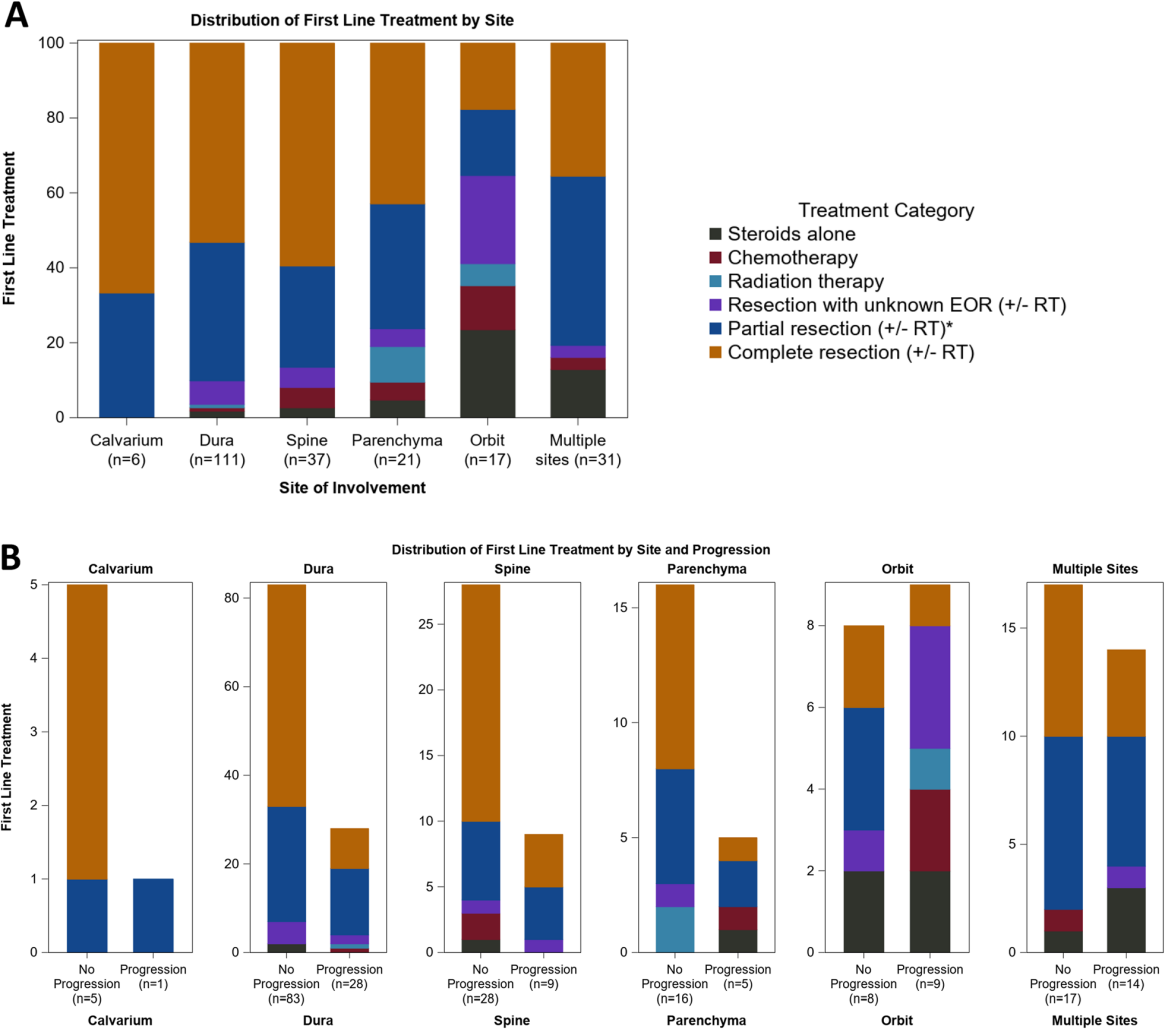
(**A**) Stacked bar charts presenting treatment modalities per site of disease involvement. (**B**) Stacked bar charts, paired per site of disease, demonstrate distribution of treatment in those with versus without subsequent progression. RT; radiation. EOR; extent of resection. *Four patients in the partial resection category also received chemotherapy

First-line treatment involved surgical resection in 200 (89%) patients (complete resection (N = 108; 48.2%), partial resection (77; 34.4%), resection of unspecified extent (15; 6.7%)), steroid monotherapy in 12 (5.4%), chemotherapy in 7 (3.1%), radiation in 4 (1.8%), and observation alone in 1 (0.5%) (Fig. 2). Best radiographic responses to all first-line treatments were CR or PR in 153 (68.3%), SD in 37 (16.5%), POD in 27 (12.1%), and were not reported on 7 patients (3.1%). Progression of disease following first-line treatment was documented in 66 patients (29.5%) and not documented in the remaining 158 (70.5%) patients. Progression-free survival (PFS) from the time of treatment was documented in 62 of the 66 with progression, with a median time to progression of 12 months. The median progression-free follow up was documented for the 151 out of the 158 patients without progression at 12 months (range 0–120 months). Of the 66 patients who progressed after first-line treatment, 25 had an initial CR or PR to first-line treatment, 8 had SD, and 7 did not have documented best radiographic response prior to progression. Progression after response to first-line treatment was observed most frequently in the setting of orbital disease (9/17, 52.9%), followed by multi-focal disease (14/32, 43.8%), dural disease (28/111, 25.2%), spine disease (9/37, 24.3%), parenchymal disease (5/21, 23.8%), and calvarial disease (1/6, 16.7%). Response and subsequent progression following each non-surgical treatment modality is presented in Table 2, and progression following individual treatment modalities, per affected site, is presented in Fig. 2.

**Table 2:** Best response for patients without resection at first line treatment

Treatment	N	Best Response				Subsequent Progression
		Complete response N (%)	Partial response N (%)	Stable disease N (%)	Progression of disease N (%)	N (%)
Observation only	1	0 (0)	0 (0)	1 (100)	0 (0)	0 (0)
Steroids alone	12	1 (8)	1 (8)	5 (42)	5 (42)	6 (50)
Chemo ± steroids	7	1 (14)	2 (29)	3 (43)	1 (14)	4 (57)
RT ± steroids	4	0 (0)	1 (25)	2 (50)	1 (25)	2 (50)
Totals	24	2	4	11	7	12

### Responses following first-line treatments involving resection

**Fig. 3:**
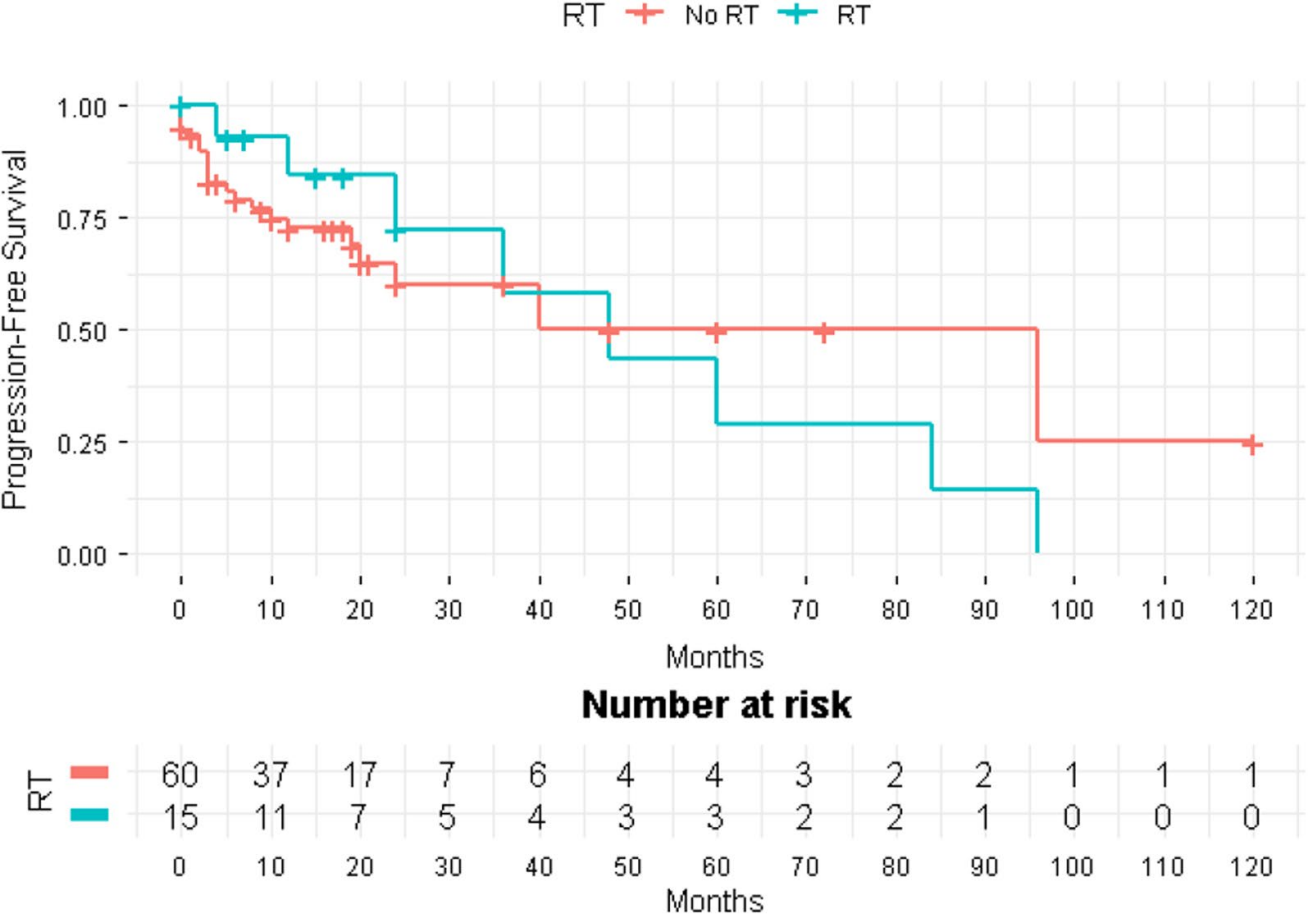
Kaplan–Meier curves comparing progression-free survival (PFS) between those receiving radiotherapy (RT) versus no radiotherapy following partial resection

Of the 200 patients who underwent resection as part of first-line treatment, 54 (27%) had subsequent progression of disease. 19/108 (17.6%) had progression following complete resection, 28/77 (36.4%) following partial resection, and 7/15 (46.7%) following resection of unknown extent. Progression following resection occurred in patients with multiple sites of disease in 11 of 26 (42.3%) cases, with orbital disease in 4 of 10 (40%), with spine disease in 9 of 34 (26.5%), with dural disease in 26 of 107 (24.3%), with parenchymal disease in 3 of 17 (17.6%), and with calvarial disease in 1 of 6 (16.7%). Altogether, 43 of 174 (24.7%) patients with unifocal disease had recurrence following surgery. Frequency of progression according to site of disease, extent of resection, and receipt of post-operative radiotherapy and/or chemotherapy are presented in Table 3. Out of 108 patients who received a complete resection as a first line treatment, 6 (5.6%) were followed with radiation and 102 (94.4%) were not. Those undergoing complete resection and radiation therapy had 4/6 (66.7%) documented cases of subsequent progression, versus 15/102 (14.7%) among those receiving complete resection only; this small sample size did not allow for statistical comparison of PFS. On the other hand, out of the 77 patients who underwent partial resection, 15 (19.5%) were followed with radiation and 62 (80.5%) were not. Eight out of 15 (53.3%) patients who underwent a partial resection and radiation had progression versus 20 out of 62 (32.3%) not receiving radiation. Comparing progression-free survival between patients who received radiotherapy (RT) following their partial resection to those who did not, no significance in their progression free survival was observed (Fig. 3). Two patients received chemotherapy in addition to partial resection and radiation, both with subsequent progression, and two received chemotherapy following partial resection without radiation, and neither had subsequent progression.

**Table 3:** Progression of neurologic Rosai–Dorfman disease by extent of surgical resection and receipt of radiation therapy (N = 200)

Extent of resection	Calvarium (N = 6)		Dura (N = 107)		Spine (N = 34)		Parenchyma (N = 17)		Orbit (N = 10)		Multiple sites (N = 26)		All sites combined (N = 200)	
	N (%)	POD N (%)	N (%)	POD N (%)	N (%)	POD N (%)	N (%)	POD N (%)	N (%)	POD N (%)	N (%)	POD N (%)	Total N (%)	POD N (%)
Complete resection														
With radiotherapy	0 (0)	0 (0)	1 (0.1)	1 (100)	1 (3)	0 (0)	1 (6)	1 (100)	0 (0)	0 (0)	3 (12)	2 (67)	6 (3)	4
Without radiotherapy	4 (67)	0 (0)	58 (54)	8 (14)	21 (62)	4 (19)	8 (47)	0 (0)	3 (30)	1 (33)	8 (31)	2 (25)	102 (51)	15
Partial resection														
With radiotherapy	1 (17)	0 (0)	9 (8)	4 (44)	0 (0)	0 (0)	0 (0)	0 (0)	0 (0)	0 (0)	5 (19)	4 (80)	15 (8)	8
Without radiotherapy	1 (17)	1 (100)	32 (30)	11 (34)	10 (29)	4 (40)	7 (41)	2 (29)	3 (30)	0 (0)	9 (35)	2 (29)	62 (31)	20
Unknown	0 (0)	0 (0)	7 (7)	2 (29)	2 (6)	1 (50)	1 (6)	0 (0)	4 (40)	3 (75)	1 (4)	1 (100)	15 (8)	7
Total	6	1 (16.7%)	107	26 (24.3%)	34	9 (26.5%)	17	3 (17.6)	10	4 (40.0%)	26	11 (42.3%)	200	54

### Responses to first-line chemotherapy

Chemotherapy was implemented as first-line treatment in 11/224 (4.9%) patients, 4 in combination with other treatments and 7 as monotherapy and led to various responses. Chemotherapy was adopted following partial resection and radiation in two patients, one of whom was treated with vinblastine and had progressive disease 12 months after treatment with no reported initial best radiographic response. The second patient received methotrexate, mercaptopurine, and vincristine achieving PR for 84 months. Two other patients received chemotherapy following partial resection alone, one of whom was treated with chlorambucil achieving CR for 24 months, and another one received methotrexate and mercaptopurine achieving SD for 12 months. Chemotherapy was given as monotherapy for the remaining 7 patients. Two patients were treated with vinblastine monotherapy, one of whom achieved PR that was sustained for 4 months without further follow up noted. The second had SD for an unknown duration followed by progression of disease. One of 2 patients treated with cladribine had SD for 24 months before having a documented relapse, and the other achieved CR for 9 months. Two patients were treated with the combination of cyclophosphamide and vincristine, one with immediate progression of disease and the other was reported to have sustained PR for 11 months without relapse. Methotrexate, mercaptopurine, and vinblastine were given in one patient achieving SD for unknown duration before documented progression.

### Second-line treatment

*S*econd-line treatment was documented in detail in 47 (21%) patients (Tables 4, 5). These treatments involved surgical resection in 23 (48.9%) patients, 3 of these receiving chemotherapy in addition to resection; complete resection with or without radiation (N = 6, 12.8%), partial resection with or without radiation (8, 17%), and resection of unspecified extent with or without radiation (9, 19.2%). Non-surgical treatments were radiation (10, 21.3%), chemotherapy (8, 17%), steroid monotherapy (5, 10.6%), and the combination of chemotherapy and radiation (1; 2.1%). Best radiographic responses to second-line therapies were CR or PR in 18 of 47 (38.3%) of cases, stable disease in 8 (17.0%), and POD in 6 (12.8%). Radiographic response was not reported in 15 (31.9%). Second line treatments had eventual progression of disease in 10 patients (21.3%). Time of progression was documented in 9 of 10 patients at a median of 3 months (range 0–20 months). The remaining 37 patients had no documented eventual progression of disease. The period of follow up was documented in 19 of them at a median of 16 months (range 0–84 months).

**Table 4:** Best radiographic responses to second line treatments

Best radiographic response	Chemo ± steroids N = 8	Rad ± steroids N = 10	Chemo + RT N = 1	Steroids alone N = 5
CR	2 (25)	1 (10)	0 (0)	3 (60)
PR	0 (0)	4 (40)	0 (0)	1 (20)
SD	2 (25)	1 (10)	1 (100)	0 (0)
POD	0 (0)	2 (20)	0 (0)	1 (20)
Unknown	4 (50)	2 (20)	0 (0)	0 (0)

**Table 5:** Subsequent progression of neurologic Rosai–Dorfman disease by extent of surgical resection

Subsequent progression	Complete EOR ± RT N = 6	Partial EOR ± RT N = 8	Unknown EOR ± RT N = 9
Yes	0 (0)	2 (25)	3 (33)
No	4 (67)	4 (50)	4 (44)
Unknown	2 (33)	2 (25)	2 (22)

Chemotherapy was implemented as a part of second line treatment in 12 (25.5%) patients. It was combined with surgical resection in 3 patients, radiotherapy in 1 patient, and used alone in the remaining 8 patients. Best radiographic response was documented in 7 patients who received chemotherapy second line. A patient who received a resection of an unknown extent followed by cyclophosphamide, vincristine, and doxorubicin achieved CR for 84 months. Two other patients treated with azathioprine monotherapy and a combination of cyclophosphamide, vincristine, and doxorubicin also achieved CR and were followed for 35 and 84 months, respectively. The remaining 4 patients achieved SD; one was treated with radiation and temozolomide and followed for 36 months. One was treated with cladribine, one with melphalan, with no reported duration of follow up of their stable disease. The fourth received a resection of an unknown extent followed by vinblastine and mercaptopurine and no duration of follow up of their stable disease was reported. Radiographic response was not reported in the other 5 patients whose second line treatments included chemotherapy and their regimens included vinblastine following a complete resection, vincristine monotherapy, cyclophosphamide and etoposide, etoposide and intrathecal methotrexate, and cytarabine.

### Third- and later line treatment

Six (2.7%) patients were documented to have received third-line therapy. Two patients were treated with radiotherapy (RT) which was the second course of RT to one of them who was then documented to have achieved PR of unknown duration and the third course of RT for the other only to have progression of disease 4.5 months later. The remaining 4 patients were treated with chemotherapy; two of them with cyclophosphamide and vincristine; achieving SD for unknown duration for one patient and POD after 6 months for the other. Cytarabine was given in the third patient achieving SD of unknown duration before eventual progression. The last patient received a combination of methotrexate, vinblastine, and mercaptopurine achieving SD for 4 months before progression. All three patients who were documented to go on to having a fourth line of therapy had orbital disease. One of them achieved CR of their orbital disease for 17 months with single-agent clofarabine. Another was re-resected again, only to progress 6 months later and undergo their first course of RT achieving SD for 14 months. The third, had PR for 7 months also to their first course of radiation.

## Discussion

In this systematic review, we synthesize the published empiric literature about neurologic involvement of RDD. We identified 154 articles with clinical data from 224 patients; 128 patients were contained in single case reports and the largest series contained 10 patients. The most common presenting symptoms were headache (45.1%), focal deficits (32.6%), and visual symptoms (32.1%). Approximately half (49.6%) presented with dural infiltration, and less common sites of disease were the spine (16.5%), brain parenchyma (9.4%), and multiple sites (14.3%). CR or PR was achieved by first-line treatment in 68.3% of cases, and 25 (16.3%) of those with responses had subsequent disease progression. CR or PR was achieved by second-line treatment in only 18 (38.3)% of cases, and across the patient population and lines of therapy, responses to systemic chemotherapy were modest.

First, our literature search highlights the dearth of rigorous data about neurologic RDD. The data presented here were derived entirely from case reports and retrospective series of 10 or fewer patients, and none from prospective trials. Second, our data demonstrate the wide spectrum of presenting clinical symptoms in neurologic RDD which included seizures, cranial neuropathies, cerebellar dysfunction, diabetes insipidus, and other neuro-endocrine abnormalities. Cerebellar dysfunction and diabetes insipidus are classically associated with Erdheim-Chester disease (ECD) and Langerhans cell histiocytosis (LCH) but are rarely thought to be referable to RDD. A recent study of 30 patients with neurologic ECD demonstrated a wide and heterogeneous array of clinical presentations of that disease [15], and our series similarly raises awareness of the broad spectrum of neurologic RDD. By contrast, the largest study of neurologic ECD patients from the Pitie-Salpetriere [16] described both neurovascular and neurodegenerative phenotypes ECD, and these entities do not emerge in our review of RDD. Likewise, no clinical cases reminiscent of the neurodegenerative entities rarely observed as a late consequence of LCH [17] were documented in RDD. This comprehensive review of the existing literature compels clinicians to have a more inclusive clinical perspective of neurologic RDD in light of its varied symptomatology and presentations, however there are features that remain more characteristic of other neurohistiocytoses.

In terms of sites of disease, the dura was the most common site of RDD involvement, followed by the spine, brain parenchyma, orbits, and calvarium. There was a subset of patients who presented with a multi-focal involvement of the nervous system as well. This distribution of neurohistiocytic involvement differs from that described in ECD which parenchymal brain lesions are the most common, including the posterior fossa and cerebellum, then calvarial lesions, then dura, and then orbits. Large observational imaging studies of neurologic LCH demonstrate a different pattern of infiltration as well, with most common infiltration of osseous structures, hypothalamic-pituitary axis (HPA) and posterior fossa, and only rare reports of meningeal disease [18, 19]. Other non-LCH disorders which rarely affect the nervous system, such as juvenile xanthogranuloma, have not been characterized in any large series but have been documented to involve numerous structures including the dura, cerebral hemispheres, posterior fossa, HPA, cranial nerves, deep grey structures, and base of skull [20–22]. Regarding molecular characteristics, the articles included in our review presented no mutational data about mitogen-activated protein kinase (MAPK) pathway mutations identified in RDD tissue. Recent studies have identified *NRAS*, *KRAS*, *MAP2K1*, and *ARAF* mutations in RDD patients without documented neurologic disease [7, 23]. The great majority of the cases and series reviewed in our study were published prior to the molecular era of histiocytosis, and therefore dedicated mutational studies of neurologic RDD have yet to be performed. In the 30-patient neurologic ECD series mentioned above, diverse kinase mutations were identified within *BRAF*, *RAS* isoforms, *MAP2K1*, and others. Future investigation may shed like upon molecular features of RDD neurohistiocytosis.

There are several observations to be made regarding treatments, responses, and subsequent progression. First, 54 (27%) of 200 patients undergoing resection for neurologic RDD had subsequent progression, including 43 of 174 (24.7%) with unifocal disease. This finding of roughly one-quarter having disease progression stands in contrast to the prevailing notion that unifocal neurologic lesions are most likely to be definitively managed with resection [6, 24]. Furthermore, despite the limitation presented by the numbrer of cases with reported follow up, our data suggests that radiotherapy following partial resection is not necessarily effective in preventing RDD recurrence. Another striking finding is the scarcity of empiric data regarding systemic steroids and chemotherapy for neurologic RDD, as well as the generally unfavorable responses that they conferred. As first-line treatment, steroid monotherapy was given to 12 patients with 7 CR, PR, or SD, and 5 POD as best response; progression was observed in 1 of the 7 with CR, PR, or SD. First-line chemo monotherapy led to 6 CR, PR, or SD among 7 patients treated, 3 with subsequent progression; as second-line monotherapy, chemotherapy led to 2 CR and 2 SD among 8 treated. Among all patients reviewed, the only chemotherapies leading to PR or CR in any instances were vinblastine, combined methotrexate/mercaptopurine/vincristine, clofarabine, chlorambucil, combined cyclophosphamide/vincristine/doxorubicin, and azathioprine. Of note, these were all single cases. This finding of suboptimal response to chemotherapy is resonant with what was observed in a series of 30 patients with ECD, for whom “conventional” (i.e. chemotherapeutic and immunosuppressive) treatments were generally not efficacious (20% PR by MRI, 31% CR or PR by positron-emission tomography (PET)). By contrast, chemotherapies have been found to be more effective for neurologic LCH, including vinblastine in one retrospective series of 20 pediatric patients [25] in which 15 (75%) had CR or PR. Cladribine was observed to lead to response without relapse in 17/17 pediatric patients with CNS LCH in one French series [26], as well as 12/12 responses in a series of mixed adults and children with CNS LCH in another series [27]. Finally, this review provides very little data regarding radiotherapy only for neurologic RDD with 4 instances as first-line treatment (1 PR, 2 SD, 1 POD) and 10 as second-line treatment (1 CR, 4 PR, 1 SD, 2 POD, 2 unknown), but it would seem that further study of this treatment may be merited.

This study has limitations. First, we reviewed only articles in English which restricted our sample of published cases. Second, we were unable to independently confirm the diagnosis of RDD by slide review as this would not be possible for this number of cases occurring across decades and numerous institutions; as a result, it is possible that a subset of the included cases may involve a misdiagnosis as RDD and other histiocytoses are complex and multifaceted diagnoses. Also, information was lacking about evaluation for RDD-associated disorders such as autoimmune disease and other neoplasms which may have affected treatments and responses. Mixed forms of histiocytosis, such as RDD lesions happening in the setting of ECD, have only recently been described [28], and this entity was not accounted for by these earlier cases. Last, we chose to include articles with a complete clinical presentation including a treatment method and radiographic response documentation; there is an inherent bias in examining cases that have been published in that they may have been more likely to involve comprehensive evaluation and effective treatment. In this way we acknowledge that these cases may not be entirely representative of the spectrum of neurologic RDD.

Despite these limitations, we believe that we have characterized and presented the existing published knowledge about RDD neurohistiocytosis, including its diversity of clinical presentations and management challenges. More effective systemic therapies are needed for patients with multifocal disease and those with unifocal disease with progression following local treatments. The role of targeted therapies for MAPK pathway mutated neurologic RDD requires further characterization. Future collaborative research may shed further light on neurologic manifestations of RDD and other histiocytic disorders and improve outcomes for these patients.

## Supplementary Information

**Additional file 1:** Article list.

**Additional file 2:** Research terms.

## Data Availability

Complete list of included articles is included in the Additional file 1. Analysis data stored and available upon request, please contact authors for data requests.

## Code availability

Nonapplicable.
