# Supplementary material for: Rosai–Dorfman–Destombes disease of the nervous system: a systematic literature review
Source: Orphanet J Rare Dis. 2022 Mar 2;17:92. doi: 10.1186/s13023-022-02220-0 (PMC8889645; doi:10.1186/s13023-022-02220-0)
Supplement: Supplementary file 1 — Additional file 1: Article list. [file 13023_2022_2220_MOESM1_ESM.pdf]

Author(s)	Title	Year	Journal	Volume	Issue
Abou-Zeid, A. H., Herwadkar, A., du Plessis, D. and Gnanalingham, K. K.	Isolated extradural Rosai-Dorfman disease of the thoracic spine: a rare cause of spinal cord compression: case report	2010	Neurosurgery	67	2
Adeleye, A. O., Amir, G., Fraifeld, S., Shoshan, Y., Umansky, F. and Spektor, S.	Diagnosis and management of Rosai-Dorfman disease involving the central nervous system	2010	Neurol Res	32	6
Alimli, A. G., Oztunali, C., Boyunaga, O. L., Pamukcuoglu, S., Okur, A. and Borcek, A. O.	MRI and CT findings of isolated intracranial Rosai-Dorfman disease in a child	2016	Neuroradiol J	29	2
Al-Saad, K., Thorner, P., Ngan, B. Y., Gerstle, J. T., Kulkarni, A. V., Babyn, P., Grant, R. M., Read, S., Laxer, R. M. and Chan, H. S.	Extranodal Rosai-Dorfman disease with multifocal bone and epidural involvement causing recurrent spinal cord compression	2005	Pediatr Dev Pathol	8	5
Andriko, J. A., Morrison, A., Colegial, C. H., Davis, B. J. and Jones, R. V.	Rosai-Dorfman disease isolated to the central nervous system: a report of 11 cases	2001	Mod Pathol	14	3
Anoop, T. M., John, J., Nair, S. G. and Mathew, B. S.	Intracranial Rosai Dorfman disease	2014	J Neurosci Rural Pract	5	2
Antuna Ramos, A., Alvarez Vega, M. A., Alles, J. V., Antuna Garcia, M. J. and Meilan Martinez, A.	Multiple involvement of the central nervous system in Rosai-Dorfman disease	2012	Pediatr Neurol	46	1
Aradhana, K., Thejaswini, B., Shamsundar, A., Nanda, R., Amritham, U. and Giri, G. V.	Rosai-Dorfman Disease - Five Years Retrospective Analysis from Tertiary Cancer Center	2018	Gulf J Oncolog	1	26
Arun Kumar, Y., Yi Peng, P. and Chen Chen, X.	Intracranial rosai-dorfman disease	2014	Case Rep Radiol	2014	
Beros, V., Houra, K., Rotim, K., Zivkovic, D. J., Cupic, H. and Kosec, A.	Isolated cerebellar intraparenchymal Rosai-Dorfman disease--case report and review of literature	2011	Br J Neurosurg	25	2
Bhandari, A., Patel, P. R. and Patel, M. P.	Extranodal Rosai-Dorfman disease with multiple spinal lesions: a rare presentation	2006	Surg Neurol	65	3
Bhattacharjee, M. B., Wroe, S. J., Harding, B. N. and Powell, M.	Sinus histiocytosis with massive lymphadenopathy--isolated suprasellar involvement	1992	J Neurol Neurosurg Psychiatry	55	2
Camp, S. J., Roncaroli, F., Apostolopoulos, V., Weatherall, M., Lim, S. and Nandi, D.	Intracerebral multifocal Rosai-Dorfman disease	2012	J Clin Neurosci	19	9
Cao, X. Y., Luan, S. H., Bao, W. M., Shen, C. and Yang, B. J.	Solitary intracranial Rosai-Dorfman disease: case report and literature review	2011	J Int Med Res	39	5
Carey, M. P. and Case, C. P.	Sinus histiocytosis with massive lymphadenopathy presenting as a meningioma	1987	Neuropathol Appl Neurobiol	13	5
Cascone, P., Santamaria, S., Mercurio, A. and Polito, E.	The use of the neuronavigator in the orbital surgery of a rare case of Rosai-Dorfman disease	2004	J Craniofac Surg	15	4

Chang, Y. C., Tsai, M. H., Chen, C. L., Tsai, C. H. and Lee, A. Y.	Nasal Rosai-Dorfman disease with intracranial involvement: a case report	2003	Am J Otolaryngol	24	3
Chen, C. W., Kachramanoglou, C., Revesz, T. and Choi, D.	Rosai-Dorfman disease presenting as a thoracic intradural extramedullary spinal tumor but without extraspinal manifestations	2012	Acta Neurochir (Wien)	154	2
Chen, K. T.	Crush cytology of Rosai-Dorfman disease of the central nervous system. A report of 2 cases	2003	Acta Cytol	47	6
Chen, M. W., King, N. K., Selvarajan, S. and Low, D. C.	Benign scalp lump as an unusual presentation of extranodal Rosai-Dorfman disease	2014	Surg Neurol Int	5	
Chhabria, B. A., Nampoothiri, R. V., Nambiyar, K. and Lad, D.	A quintessential syndrome with a rare marvelling aetiology: Rosai-Dorfman disease presenting as Conus-Cauda syndrome	2018	BMJ Case Rep	2018	
Chivukula, S., Clark, K., Murdoch, G. and Engh, J.	A Singular Case of Intracranial Sinus Histiocytosis without Massive Lymphadenopathy: Isolated Rosai-Dorfman Disease of the Hypothalamus	2015	J Neurol Surg A Cent Eur Neurosurg	76	3
Clark, W. C. and Berry, A. D., 3rd	Extranodal sinus histiocytosis with massive lymphadenopathy: isolated central nervous system involvement mimicking meningioma	1996	South Med J	89	6
Cooper, S. L., Chavis, P. S., Fortney, J. A., Watkins, J. M., Caplan, M. J. and Jenrette, J. M., 3rd	A case of orbital Rosai-Dorfman disease responding to radiotherapy	2008	J Pediatr Hematol Oncol	30	10
Das, S., Biswas, A., Roy, S., Sable, M. N., Singh, D., Jana, M., Sharma, M. C. and Julka, P. K.	Recurrent intracranial Rosai-Dorfman disease: Management of a challenging case	2017	Asian J Neurosurg	12	3
Deodhare, S. S., Ang, L. C. and Bilbao, J. M.	Isolated intracranial involvement in Rosai-Dorfman disease: a report of two cases and review of the literature	1998	Arch Pathol Lab Med	122	2
Di Rocco, F., Garnett, M. R., Puget, S., Pueyerredon, F., Roujeau, T., Jaubert, F. and Sainte-Rose, C.	Cerebral localization of Rosai-Dorfman disease in a child. Case report	2007	J Neurosurg	107	2 Suppl
Dran, G., Rasendrarijao, D., Vandenbos, F. and Paquis, P.	Rosai-Dorfman disease causing spinal cord compression: case report	2008	Neurosurgery	62	4
Fortea, J., Compta, Y., Valldeoriola, F., Tolosa, E., Rey, M. J., Gaston, F. and Ribalta, T.	Fatal worsening of late-onset cerebellar ataxia with neuronal intranuclear inclusions due to superimposed meningeal Rosai-Dorfman disease	2008	Mov Disord	23	10
Fukushima, T., Yachi, K., Ogino, A., Ohta, T., Watanabe, T., Yoshino, A. and Katayama, Y.	Isolated intracranial Rosai-Dorfman disease without dural attachment--case report	2011	Neurol Med Chir (Tokyo)	51	2

Geara, A. R., Ayoubi, M. A., Achram, M. C. and Chamseddine, N. M.	Rosai-Dorfman disease mimicking neurofibromatosis: case presentation and review of the literature	2004	Clin Radiol	59	7
Ghosal, N., Murthy, G., Visvanathan, K., Sridhar, M. and Hegde, A. S.	Isolated intracranial Rosai Dorfman disease masquerading as meningioma: a case report	2007	Indian J Pathol Microbiol	50	2
Goldberg, S., Mahadevia, P., Lipton, M. and Rosenbaum, P. S.	Sinus histiocytosis with massive lymphadenopathy involving the orbit: reversal of compressive optic neuropathy after chemotherapy	1998	J Neuroophthalmol	18	4
Griffiths, S. J., Tang, W., Parameswaran, R., Kelsey, A. and West, C. G.	Isolated intracranial Rosai-Dorfman disease mimicking meningioma in a child	2004	Br J Neurosurg	18	3
Gupta, D. K., Suri, A., Mahapatra, A. K., Mehta, V. S., Garg, A., Sarkar, C. and Ahmad, F. U.	Intracranial Rosai-Dorfman disease in a child mimicking bilateral giant petroclival meningiomas: a case report and review of literature	2006	Childs Nerv Syst	22	9
Haas, R. J., Helmig, M. S. and Meister, P.	Sinus histiocytosis with massive lymphadenopathy and epidural involvement	1981	Haematol Blood Transfus	27	
Hadjipanayis, C. G., Bejjani, G., Wiley, C., Hasegawa, T., Maddock, M. and Kondziolka, D.	Intracranial Rosai-Dorfman disease treated with microsurgical resection and stereotactic radiosurgery. Case report	2003	J Neurosurg	98	1
Hashimoto, K., Kariya, S., Onoda, T., Ooue, T., Yamashita, Y., Naka, K., Okano, M. and Nishizaki, K.	Rosai-Dorfman disease with extranodal involvement	2014	Laryngoscope	124	3
Hinduja, A., Aguilar, L. G., Steineke, T., Nochlin, D. and Landolfi, J. C.	Rosai-Dorfman disease manifesting as intracranial and intraorbital lesion	2009	J Neurooncol	92	1
Hollon, T., Camelo-Piragua, S. I., McKean, E. L., Sullivan, S. E. and Garton, H. J.	Surgical Management of Skull Base Rosai-Dorfman Disease	2016	World Neurosurg	87	
Hollowell, J. P., Wolfla, C. E., Shah, N. C., Mark, L. P. and Whittaker, M. H.	Rosai-Dorman disease causing cervical myelopathy	2000	Spine (Phila Pa 1976)	25	11
Hong, C. S., Starke, R. M., Hays, M. A., Mandell, J. W., Schiff, D. and Asthagiri, A. R.	Redefining the Prevalence of Dural Involvement in Rosai-Dorfman Disease of the Central Nervous System	2016	World Neurosurg	90	
Huang, B. Y., Liu, H. L. and Yu, C. J.	Isolated Intramedullary Spinal Rosai-Dorfman Disease: A Case Report and Literature Review	2016	World Neurosurg	88	
Huang, B. Y., Zhang, H., Zong, W. J. and Sun, Y. H.	Rosai-Dorfman Disease of Rare Isolated Spinal Involvement: Report of 4 Cases and Literature Review	2016	World Neurosurg	85	

Huang, B. Y., Zong, M., Zong, W. J., Sun, Y. H., Zhang, H. and Zhang, H. B.	Intracranial Rosai-Dorfman disease	2016	J Clin Neurosci	32	
Huang, Y. C., Tan, H. Y., Jung, S. M., Chuang, W. Y., Chuang, C. C., Hsu, P. W. and Chang, C. N.	Spinal epidural Rosai-Dorfman disease preceding by relapsing uveitis: a case report with literature review	2007	Spinal Cord	45	9
Imada, H., Sakatani, T., Sawada, M., Matsuura, T., Fukushima, N. and Nakano, I.	A lethal intracranial Rosai-Dorfman disease of the brainstem diagnosed at autopsy	2015	Pathol Int	65	10
Johnston, J. M., Limbrick, D. D., Ray, W. Z., Brown, S., Shimony, J. and Park, T. S.	Isolated cerebellar Rosai-Dorfman granuloma mimicking Lhermitte-Duclos disease. Case report	2009	J Neurosurg Pediatr	4	2
Joshi, S. S., Joshi, S., Muzumdar, G., Turel, K. E., Shah, R. M., Ammbulkar, I., Hussain, M. M. and Choudhari, K. A.	Cranio-spinal Rosai Dorfman disease: case series and literature review	2017	Br J Neurosurg		
Juric, G., Jakic-Razumovic, J., Rotim, K. and Zarkovic, K.	Extranodal sinus histiocytosis (Rosai-Dorfman disease) of the brain parenchyma	2003	Acta Neurochir (Wien)	145	2
Kattner, K. A., Stroink, A. R., Roth, T. C. and Lee, J. M.	Rosai-Dorfman disease mimicking parasagittal meningioma: case presentation and review of literature	2000	Surg Neurol	53	5
Katz, D. S., Poe, L. B. and Corona, R. J., Jr.	Sinus histiocytosis with massive lymphadenopathy: a case of simultaneous upper respiratory tract and CNS disease without lymphadenopathy	1993	AJNR Am J Neuroradiol	14	1
Kelly, W. F., Bradey, N. and Scoones, D.	Rosai-Dorfman disease presenting as a pituitary tumour	1999	Clin Endocrinol (Oxf)	50	1
Kidd, D. P., Revesz, T. and Miller, N. R.	Rosai-Dorfman disease presenting with widespread intracranial and spinal cord involvement	2006	Neurology	67	9
Kim, D. Y., Park, J. H., Shin, D. A., Yi, S., Ha, Y., Yoon, D. H. and Kim, K. N.	Rosai-dorfman disease in thoracic spine: a rare case of compression fracture	2014	Korean J Spine	11	3
Kim, M., Provias, J. and Bernstein, M.	Rosai-Dorfman disease mimicking multiple meningioma: case report	1995	Neurosurgery	36	6
Kim, S. I., Kim, S. H., Cho, H. J., Kim, H., Chung, C. K., Choi, S. H. and Park, S. H.	Mass-forming primary angiitis of central nervous system with Rosai-Dorfmann disease-like massive histiocytosis with emperipolesis	2015	Pathol Int	65	8
Kitai, R., Llena, J., Hirano, A., Ido, K., Sato, K. and Kubota, T.	Meningeal Rosai-Dorfman disease: report of three cases and literature review	2001	Brain Tumor Pathol	18	1

Konca, C., Ozkurt, Z. N., Deger, M., Aki, Z. and Yagci, M.	Extranodal multifocal Rosai-Dorfman disease: response to 2-chlorodeoxyadenosine treatment	2009	Int J Hematol	89	1
Konishi, E., Ibayashi, N., Yamamoto, S. and Scheithauer, B. W.	Isolated intracranial Rosai-Dorfman disease (sinus histiocytosis with massive lymphadenopathy)	2003	AJNR Am J Neuroradiol	24	3
Kraeft, S. K., Honig, M. and Krishnamurthy, S.	Emperipolesis in the cerebrospinal fluid from a patient with Rosai-Dorfman disease	2008	Diagn Cytopathol	36	1
Krishnakumar, S., Babu, K., Das, D. and Biswas, J.	A clinicopathologic study of sinus histiocytosis with massive lymphadenopathy mimicking an optic nerve tumor	2003	J Pediatr Ophthalmol Strabismus	40	3
Krishnamoorthy, V., Parmar, C. F. and Panikar, D.	Isolated intracranial Rosai Dorfman disease	2011	Neurol India	59	3
Le Guenno, G., Galicier, L., Uro-Coste, E., Petitcolin, V., Rieu, V. and Ruivard, M.	Successful treatment with azathioprine of relapsing Rosai-Dorfman disease of the central nervous system	2012	J Neurosurg	117	3
Li, Y., Sun, H., Zhang, Y. and Liu, W.	Isolated intracranial Rosai-Dorfman disease presenting as mental deterioration	2012	Clin Neurol Neurosurg	114	7
Li, Z., Zhou, C., Chen, G. and Bao, Y.	Intracranial Rosai-Dorfman disease involving the cavernous sinus: a case report and review of the literature	2018	World Neurosurg		
Lu, C. H., Chang, K. C., Lee, E. J., Chuang, M. T. and Chang, R. S.	Intracranial Rosai-Dorfman disease with unusual transcranial extension	2012	J Neuroimaging	22	3
Luo, Z., Zhang, Y., Zhao, P., Lu, H., Yang, K., Zhang, Y. and Zeng, Y.	Characteristics of Rosai-Dorfman Disease Primarily Involved in the Central Nervous System: 3 Case Reports and Review of Literature	2017	World Neurosurg	97	
Maiti, T. K., Gangadharan, J., Mahadevan, A., Arivazhagan, A., Chandramouli, B. A. and Shankar, S. K.	Rosai-Dorfman disease presenting as cervical extradural lesion: a case report with review of literature	2011	Neurol India	59	3
Maratos, E. C., Bridges, L. R., MacKinnon, A. D., Madigan, J. B., Atra, A. and Martin, A. J.	Isolated intracranial Rosai-Dorfman disease in a child, a case report and review of the literature	2014	Childs Nerv Syst	30	9
McPherson, C. M., Brown, J., Kim, A. W. and DeMonte, F.	Regression of intracranial rosai-dorfman disease following corticosteroid therapy. Case report	2006	J Neurosurg	104	5
Mitra, S., Kundu, S., Majumdar, A. and Pattari, S. K.	A young male with paraplegia, massive cervical adenopathy and nondiagnostic biopsy specimens	2007	J Assoc Physicians India	55	
Morandi, X., Godey, B., Riffaud, L., Heresbach, N. and Brassier, G.	Isolated Rosai-Dorfman disease of the fourth ventricle. Case illustration	2000	J Neurosurg	92	5

Nassif, S. and Boulos, F.	Extranodal (dural) Rosai-Dorfman disease radiologically and histologically mimicking meningioma: a case report	2015	Anal Quant Cytopathol Histpathol	37	2
Nemir, J., Trninic, I., Duric, K. S., Jakovcevic, A., Mrak, G. and Paladino, J.	Extranodal right-optic nerve Rosai-Dorfman disease: A rare localization case report	2016	Surg Neurol Int	7	Suppl 44
Osenbach, R. K.	Isolated extranodal sinus histiocytosis presenting as an intramedullary spinal cord tumor with paraplegia. Case report	1996	J Neurosurg	85	4
Parmar, V., Seward, C., Huho, A., Qian, J., Gandhi, R. and Pilitsis, J. G.	Rosai-Dorfman disease presenting as cervical radiculopathy	2013	Clin Neurol Neurosurg	115	6
Petzold, A., Thom, M., Powell, M. and Plant, G. T.	Relapsing intracranial Rosai-Dorfman disease	2001	J Neurol Neurosurg Psychiatry	71	4
Pless, M. and Chang, B. M.	Rosai-Dorfman disease. Extranodal sinus histiocytosis in three co-existing sites. A case report	2003	J Neurooncol	61	2
Purav, P., Ganapathy, K., Mallikarjuna, V. S., Annapurneswari, S., Kalyanaraman, S., Reginald, J., Natarajan, P., Bapu, K. R. and Balamurugan, M.	Rosai-Dorfman disease of the central nervous system	2005	J Clin Neurosci	12	6
Raslan, O. A., Schellingerhout, D., Fuller, G. N. and Ketonen, L. M.	Rosai-Dorfman disease in neuroradiology: imaging findings in a series of 10 patients	2011	AJR Am J Roentgenol	196	2
Resnick, D. K., Johnson, B. L. and Lovely, T. J.	Rosai-Dorfman disease presenting with multiple orbital and intracranial masses	1996	Acta Neuropathol	91	5
Reynolds, M. R., Sweeney, K. J., Crilly, S. M., Farrell, M., Jaffe, E. S. and Javadpour, M.	Rapid, de novo development of isolated intracranial rosai-dorfman disease: A case report	2014	Br J Neurosurg		
Richardson, T. E., Wachsmann, M., Oliver, D., Abedin, Z., Ye, D., Burns, D. K., Raisanen, J. M., Greenberg, B. M. and Hatanpaa, K. J.	BRAF mutation leading to central nervous system rosai-dorfman disease	2018	Ann Neurol		
Rivera, D., Perez-Castillo, M., Fernandez, B. and Stoeter, P.	Long-term follow-up in two cases of intracranial Rosai-Dorfman Disease complicated by incomplete resection and recurrence	2014	Surg Neurol Int	5	
Roy, C., Saha, A., Roy, S. and Ghosh, A.	Extranodal Rosai-Dorfman Disease presenting as spinal extradural lesion: a case report with a review of the literature	2012	J Cancer Res Ther	8	4
Russo, N., Giangaspero, F., Beccaglia, M. R. and Santoro, A.	Intracranial dural histiocytosis	2009	Br J Neurosurg	23	4

Said, R., Abi-Fadel, F., Talwar, J., Attallah, J. P. and Dilawari, A.	Intracranial rosai-dorfman: a clinical challenge	2011	Neurologist	17	2
Sakai, K., Koike, G., Seguchi, K. and Nakazato, Y.	Sinus histiocytosis with massive lymphadenopathy: a case of multiple dural involvement	1998	Brain Tumor Pathol	15	2
Sandoval-Sus, J. D., Sandoval-Leon, A. C., Chapman, J. R., Velazquez-Vega, J., Borja, M. J., Rosenberg, S., Lossos, A. and Lossos, I. S.	Rosai-Dorfman disease of the central nervous system: report of 6 cases and review of the literature	2014	Medicine (Baltimore)	93	3
Sciacca, S., Barkas, K., Heptinstall, L., McNamara, C. and Shetty, R.	Rosai-Dorfman disease with spinal cord compression: a diagnostic challenge	2015	Eur Spine J	24 Suppl 4	
Seyednejad, F., Tubbs, R. S., Shoja, M. M., Daghigi, M. H. and Oakes, W. J.	Presumed recurrence of intracranial Rosai-Dorfman disease as a cervical spine tumor	2007	Acta Neurochir (Wien)	149	4
Sharma, M. S., Padua, M. D. and Jha, A. N.	Rosai-Dorfman disease mimicking a sphenoid wing meningioma	2005	Neurol India	53	1
Shaver, E. G., Rebsamen, S. L., Yachnis, A. T. and Sutton, L. N.	Isolated extranodal intracranial sinus histiocytosis in a 5-year-old boy. Case report	1993	J Neurosurg	79	5
Simos, M., Dimitrios, P. and Philip, T.	A new clinical entity mimicking meningioma diagnosed pathologically as rosai-dorfman disease	1998	Skull Base Surg	8	2
Siu, R. C., Tan, I. L., Davidson, A. S., Robertson, A. and Fraser, C. L.	Clinical Reasoning: compressive optic neuropathy secondary to intracranial Rosai-Dorfman disease	2015	Neurology	85	12
Stojkovic, T., de Seze, J., Maurage, C. A., Rose, C., Hache, J. C. and Vermersch, P.	Atypical form of non-Langerhans histiocytosis with disseminated brain and leptomeningeal lesions	2000	J Neurol Neurosurg Psychiatry	69	5
Sundaram, C., Uppin, S. G., Prasad, B. C., Sahu, B. P., Devi, M. U., Prasad, V. S. and Purohit, A. K.	Isolated Rosai Dorfman disease of the central nervous system presenting as dural-based and intraparenchymal lesions	2005	Clin Neuropathol	24	3
Symss, N. P., Cugati, G., Vasudevan, M. C., Ramamurthi, R. and Pande, A.	Intracranial Rosai Dorfman Disease: report of three cases and literature review	2010	Asian J Neurosurg	5	2
Tan, S., Ruan, L., Jin, K., Wang, F., Mou, J., Huang, H. and Yang, G.	Systemic Rosai-Dorfman disease with central nervous system involvement	2018	Int J Neurosci	128	2
Tanboon, J., Chaipipat, M., Wattanasirmkit, V., Wongtabtim, W., Shuangshoti, S. and Bunyaratavej, K.	Squash cytology of Rosai-Dorfman disease in the sellar region	2003	Acta Cytol	47	6
Tavangar, S. M., Mahta, A., Haghpanah, V. and Larijani, B.	Extranodal Rosai-Dorfman disease involving the meninges in a 79-year-old man	2006	Ann Saudi Med	26	6

Theeler, B. J., Keylock, J. B. and Yoest, S. M.	Teaching NeuroImage: isolated intracranial Rosai-Dorfman disease mimicking a meningioma	2008	Neurology	70	13
Tian, Y., Wang, J., Ge, J., Ma, Z. and Ge, M.	Intracranial Rosai-Dorfman disease mimicking multiple meningiomas in a child: a case report and review of the literature	2015	Childs Nerv Syst	31	2
Tian, Y., Wang, J., Li, M., Lin, S., Wang, G., Wu, Z., Ge, M. and Pirotte, B. J.	Rosai-Dorfman disease involving the central nervous system: seven cases from one institute	2015	Acta Neurochir (Wien)	157	9
Toh, C. H., Chen, Y. L., Wong, H. F., Wei, K. C., Ng, S. H. and Wan, Y. L.	Rosai-Dorfman disease with dural sinus invasion. Report of two cases	2005	J Neurosurg	102	3
Tubbs, R. S., Kelly, D. R., Mroczek-Musulman, E. C., Hammers, Y. A., Berkow, R. L., Oakes, W. J. and Grabb, P. A.	Spinal cord compression as a result of Rosai-Dorfman disease of the upper cervical spine in a child	2005	Childs Nerv Syst	21	11
Ture, U., Seker, A., Bozkurt, S. U., Uneri, C., Sav, A. and Pamir, M. N.	Giant intracranial Rosai-Dorfman disease	2004	J Clin Neurosci	11	5
Udono, H., Fukuyama, K., Okamoto, H. and Tabuchi, K.	Rosai-Dorfman disease presenting multiple intracranial lesions with unique findings on magnetic resonance imaging. Case report	1999	J Neurosurg	91	2
Uthamalingam, P., Mehta, S. and Duraisamy, G.	Intracranial Rosai-Dorfman disease: An rare entity with an uncommon presentation	2016	Neurol India	64	5
Varan, A., Sen, H., Akalan, N., Oguz, K. K., Saglam, A. and Akyuz, C.	Pontine Rosai-Dorfman disease in a child	2015	Childs Nerv Syst	31	6
Waldron, R. L., 2nd, Paysinger, B. D., Reynolds, J. C. and Jordan, A. E.	Histiocytosis-X: extra-hypothalamic involvement of the central nervous system	1984	Br J Radiol	57	677
Walker, R. N., Nickles, T. P., Lountzis, N. I., Jacobs, D. L. and Nawaz, N. K.	Rosai-Dorfman disease with massive intracranial involvement: asymmetric response to conservative therapy	2011	J Neuroimaging	21	2
Wan, S., Teng, X., Zhan, R., Yu, J., Gu, J. and Zhang, K.	Isolated intracranial Rosai-Dorfman disease mimicking suprasellar meningioma: case report with review of the literature	2008	J Int Med Res	36	5
Wang, E., Anzai, Y., Paulino, A. and Wong, J.	Rosai-Dorfman disease presenting with isolated bilateral orbital masses: report of two cases	2001	AJNR Am J Neuroradiol	22	7
Wang, F., Qiao, G., Lou, X., Song, X. and Chen, W.	Intracranial recurrences of Rosai-Dorfman disease in the sellar region: two illustrative cases	2011	Acta Neurochir (Wien)	153	4
Wang, Y., Gao, X., Tang, W. and Jiang, C.	Rosai-Dorfman disease isolated to the central nervous system: a report of six cases	2010	Neuropathology	30	2

Weaver, K. D., Armao, D., Wiley, J. M. and Ewend, M. G.	Histiocytic lesion mimicking intrinsic brainstem neoplasm. Case report	1999	J Neurosurg	91	6
Wenig, B. M., Abbondanzo, S. L., Childers, E. L., Kapadia, S. B. and Heffner, D. R.	Extranodal sinus histiocytosis with massive lymphadenopathy (Rosai-Dorfman disease) of the head and neck	1993	Hum Pathol	24	5
Wolfson, W. L.	Cytopathologic presentation of cerebral histiocytosis	1979	Acta Cytol	23	5
Woodcock, R. J., Jr., Mandell, J. W. and Lipper, M. H.	Sinus histiocytosis (Rosai-Dorfman disease) of the suprasellar region: MR imaging findings--a case report	1999	Radiology	213	3
Wrzolek, M. A. and Zagzag, D.	May 2002: 38-year-old man and 69-year-old woman with dural based masses	2002	Brain Pathol	12	4
Wu, L. and Xu, Y.	Rosai-Dorfman disease: a rare lesion with dura tail sign mimicking spinal meningioma	2014	Spine J	14	12
Wu, M., Anderson, A. E. and Kahn, L. B.	A report of intracranial Rosai-Dorfman disease with literature review	2001	Ann Diagn Pathol	5	2
Wu, S. Y., Ma, L. and Tsai, Y. J.	Partial removal of orbital tumor in Rosai-Dorfman disease	2004	Jpn J Ophthalmol	48	2
Xu, H., Zhang, F., Lu, F. and Jiang, J.	Spinal Rosai-Dorfman disease: case report and literature review	2017	Eur Spine J	26	Suppl 1
Yang, X., Liu, J., Ren, Y., Richard, S. A. and Zhang, Y.	Isolated intracranial Rosai-Dorfman disease mimicking petroclival meningioma in a child: Case report and review of the literature	2017	Medicine (Baltimore)	96	47
Yao, K., Li, T. F., Zhu, M. W., Duan, Z. J., Hu, Z. L., Bian, Y. and Qi, X. L.	An intramedullary cervical cord lesion in a 12-year-old girl	2013	Neuropathology	33	5
Yataco-Vicente, J. A., Araujo-Castillo, R. V. and Lopez Fuentes, M. H.	Orbital presentation of Rosai-Dorfman disease	2018	Arch Soc Esp Oftalmol		
Yip, C. C., Cheng, C. L., Poh, W. T. and Choo, C. T.	Orbital, adnexal, and unusual systemic involvement in Rosai-Dorfman disease	2002	Ophthalmic Plast Reconstr Surg	18	3
Z'Graggen, W. J., Sturzenegger, M., Mariani, L., Keserue, B., Kappeler, A. and Vajtai, I.	Isolated Rosai-Dorfman disease of intracranial meninges	2006	Pathol Res Pract	202	3
Zhang, J. T., Tian, H. J., Lang, S. Y. and Wang, X. Q.	Primary intracerebral Rosai-Dorfman disease	2010	J Clin Neurosci	17	10
Zhang, S., Huang, J. and Chen, Y.	Primary isolated intracranial Rosai-Dorfman disease: Report of a rare case and review of the literature	2018	Neurol Neurochir Pol	52	3

Zhu, F., Zhang, J. T., Xing, X. W., Wang, D. J., Zhu, R. Y., Zhang, Q., Wang, H. T. and Lang, S. Y.	Rosai-Dorfman disease: a retrospective analysis of 13 cases	2013	Am J Med Sci	345	3
James, J. and Jose, J.	Spinal extradural Rosai Dorfman disease	2017	European Journal of General Medicine	14	1
Chen, H. H., Zhou, S. H., Wang, S. Q., Teng, X. D. and Fan, J.	Factors associated with recurrence and therapeutic strategies for sinonasal rosai-dorfman disease	2012	Head and Neck-Journal for the Sciences and Specialties of the Head and Neck	34	10
Shuangshoti, S., Navalitloha, Y., Sukpanichnant, S., Unhasuta, C. and Shuangshoti, S.	Central nervous system involvement in Rosai-Dorfman disease: Report of a case with a review of the literature	1999	Neuropathology	19	3
Smith, A. M., Tawfik, T. A., Marks, J. J., Tullis, J. E., Harkey, L. H., Manucha, V. and Kaur, J.	Rosai-Dorfman Disease with progressive central nervous system involvement requiring multiple surgical intervention: A case report	2018	Interdisciplinary Neurosurgery: Advanced Techniques and Case Management	14	
Dahrouj, M., Jakobiec, F. A., Wolkow, N., Starks, V. S. and Lee, N. G.	Atypical Case of Rosai-Dorfman Disease of the Lacrimal Gland with Adjacent Bone Erosion	2018	Ocular Oncology and Pathology		
Panse, P. M., Jensen, E. A., Cummings, K. W., Jokerst, C. E., Swanson, K. L. and Gotway, M. B.	An Unusual Disorder Involving the Central Nervous System and the Thorax	2016	Clinical Pulmonary Medicine	23	6
de Oliveira Lima, G. L., da Costa, A. C., de Paula Goes, B. H. and Junior, N. P.	Double compression caused by isolated spinal Rosai-Dorfman disease	2016	Spine Journal	16	8
Mantilla, J. G., Goldberg-Stein, S. and Wang, Y.	Extranodal rosai-dorfman disease: Clinicopathologic series of 10 patients with radiologic correlation and review of the literature	2016	American Journal of Clinical Pathology	145	2
Petrushkin, H., Salisbury, J. and O'Sullivan, E.	Intralesional steroid for orbital manifestations of Rosai-Dorfman disease	2015	Clinical and Experimental Ophthalmology	43	5
Fu, X., Jiang, J. h, Tian, X. y and Li, Z.	Isolated spinal Rosai-Dorfman disease misdiagnosed as lymphoplasmacyte-rich meningioma by intraoperative histological examination	2014	Brain Tumor Pathology		
Yamauchi, T., Hayashi, T., Matsuo, T., Matsumoto, M., Kinoshita, N., Abe, K. and Nagata, I.	Intracranial rosai-Dorfman disease-a case report and a review of the literature	2012	Acta Medica Nagasakiensia	57	2

Majumdar, K., Tyagi, I., Saran, R. K., Kumar, S. and Gondal, R.	Multicentric extranodal rosai dorfman disease - A cytological diagnosis, with histological corroboration	2012	Acta Cytologica	56	2
Triana-Pérez, A. B., Sánchez-Medina, Y., Pérez-Del Rosario, P. A., Millán-Corada, A. M., Gómez-Perals, L. F. and Domínguez-Báez, J. J.	Isolated intracranial Rosai-Dorfman disease: A case report and literature review	2011	Neurocirugia	22	3
Kros, J.	Intracranial recurrences of Rosai-Dorfman disease in the sellar region: Two illustrative cases - Commentary	2011	Acta Neurochirurgica	153	4
Huang, Y. T., Ng, S. H., Ko, S. F., Wong, H. F., Chen, Y. L., Huang, M. C., Toh, C. H. and Wai, Y. Y.	Extranodal Rosai-Dorfman disease with paranasal sinuses and intracranial involvement: A case report	2009	Chinese Journal of Radiology	34	3
Ma, J., Xiao, J. and Wang, L.	Extranodal Rosai-Dorfman disease with multilevel lumbar spinal lesions: Case report	2008	Journal of Neurosurgery: Spine	9	1
Chen, S. C., Teo, B. T. and Yen, P. S.	Isolated intracranial Rosai-Dorfman disease - Report of 2 cases and review of the literature	2007	Tzu Chi Medical Journal	19	2
Leung, J. L. Y., Cheung, J. Y. L., Tan, T. C., Tang, K. W., Chan, C. M., Ho, L. C. and Chan, S. C. H.	Carotid artery occlusion in a patient with intracranial rosai-dorfman disease	2003	Journal of the Hong Kong College of Radiologists	6	4
Simko SJ1, Tran HD, Jones J, Bilgi M, Beaupin LK, Coulter D, Garrington T, McCavit TL, Moore C, Rivera-Ortegón F, Shaffer L, Stork L, Turcotte L, Welsh EC, Hicks MJ, McClain KL, Allen CE.	Clofarabine Salvage Therapy in Refractory Multifocal Histiocytic Disorders, Including Langerhans Cell Histiocytosis, Juvenile Xanthogranuloma and Rosai–Dorfman Disease	2014	Pediatr Blood Cancer.	61	3
Foucar, E., Rosai, J., Dorfman, R. F. and Brynes, R. K.	The neurologic manifestations of sinus histiocytosis with massive lymphadenopathy	1982	Neurology	32	4
