# Supplementary material for: Rosai–Dorfman–Destombes disease of the nervous system: a systematic literature review
Source: Orphanet J Rare Dis. 2022 Mar 2;17:92. doi: 10.1186/s13023-022-02220-0 (PMC8889645; doi:10.1186/s13023-022-02220-0)
Supplement: Supplementary file 2 — Additional file 2: Research terms. [file 13023_2022_2220_MOESM2_ESM.pdf]

Supplemental Data 1

Full List Of Search Terms

PubMed (Medline) Search

(Erdheim-Chester Disease[mesh] OR Histiocytic Disorders, Malignant[mesh] OR Histiocytosis[mesh:noexp] OR Histiocytosis, Langerhans-Cell[mesh] OR Histiocytosis, Non-Langerhans-Cell[mesh:noexp] OR Histiocytosis, Sinus[mesh] OR Xanthogranuloma, Juvenile[mesh] OR Chester Erdheim Disease*[tiab] OR Dendritic Cell Sarcoma*[tiab] OR Eosinophilic Granuloma[tiab] OR Eosinophilic Granulomas[tiab] OR Erdheim Chester Disease*[tiab] OR Histiocyte[tiab] OR Histiocytic[tiab] OR Histiocytoses[tiab] OR Histiocytosis[tiab] OR Langerhans Cell[tiab] OR Nevoxanthoendothelioma*[tiab] OR Rosai-Dorfman[tiab] OR Sinus Histiocytos*[tiab] OR Xanthogranuloma[tiab] OR Xanthogranulomas[tiab]) AND ("Nervous System"[Mesh] OR "Nervous System Diseases"[Mesh] OR Brain[tiab] OR Cerebell*[tiab] OR Cerebr*[tiab] OR cogniti*[tiab] OR Cranial[tiab] OR CNS[tiab] OR dysphagia*[tiab] OR headache*[tiab] OR Intracerebr*[tiab] OR Intracranial[tiab] OR Neurolog*[tiab] OR Nervous system[tiab] OR Neurodegenerat*[tiab] OR neuropath*[tiab] OR seizure*[tiab] OR Spinal cord*[tiab]) NOT (“Animals”[mesh] NOT “Humans”[mesh]) AND English[lang]

Embase.com Search

#1 'histiocytosis'/exp OR 'sinus histiocytosis'/de OR 'xanthogranuloma'/exp OR 'chester erdheim disease*':ti,ab OR 'dendritic cell sarcoma*':ti,ab OR 'eosinophilic granuloma':ti,ab OR 'eosinophilic granulomas':ti,ab OR 'erdheim chester disease*':ti,ab OR histiocyte:ti,ab OR histiocytic:ti,ab OR histiocytoses:ti,ab OR histiocytosis:ti,ab OR 'langerhans cell':ti,ab OR nevoxanthoendothelioma*:ti,ab OR 'rosai dorfman':ti,ab OR 'sinus histiocytos*':ti,ab OR xanthogranuloma:ti,ab OR xanthogranulomas:ti,ab

#2 'nervous system'/exp OR (‘neurologic disease’/exp NOT ‘lipidosis’/exp) OR Brain:ti,ab OR Cerebell*:ti,ab OR Cerebr*:ti,ab OR cogniti*:ti,ab OR Cranial:ti,ab OR CNS:ti,ab OR Intracerebr*:ti,ab OR Intracranial:ti,ab OR Neurolog*:ti,ab OR “Nervous system”:ti,ab OR Neurodegenerat*:ti,ab OR Neuropath*:ti,ab OR “Spinal cord*”:ti,ab

Web of Science (Clarivate) Search

#1 TOPIC: (“Chester Erdheim Disease*” OR “Dendritic Cell Sarcoma*” OR “Eosinophilic Granuloma” OR “Eosinophilic Granulomas” OR “Erdheim Chester Disease*” OR Histiocyte OR Histiocytic OR Histiocytoses OR Histiocytosis OR “Langerhans Cell” OR Nevoxanthoendothelioma* OR Rosai-Dorfman OR “Sinus Histiocytos*” OR Xanthogranuloma OR Xanthogranulomas)

#2 TOPIC: (Ataxia* OR Brain OR Cerebell* OR Cerebr* OR Cogniti* OR Cranial OR CNS OR Dysarthria* OR Dysphagia* OR Dystonia*[tiab] OR Headache*OR Intracerebr* OR Intracranial OR Neurolog* OR “Nervous system” OR Neurodegenerat* OR neuropath* OR seizure* OR stroke* OR “Spinal cord*”)

Supplemental Data 1

Cochrane Library (Wiley) Search

#1 (“Chester Erdheim Disease*” OR "Dendritic Cell Sarcoma*" OR “Eosinophilic Granuloma” OR “Eosinophilic Granulomas” OR “Erdheim Chester Disease*” OR Histiocyte OR Histiocytic OR Histiocytoses OR Histiocytosis OR “Langerhans Cell” OR Nevoxanthoendothelioma* OR Rosai-Dorfman OR “Sinus Histiocytos*” OR Xanthogranuloma OR Xanthogranulomas):ti,ab

#2 (Ataxia* OR Brain OR Cerebell* OR Cerebr* OR Cogniti* OR Cranial OR CNS OR Dysarthria* OR Dysphagia* OR Dystonia* OR Headache*OR Intracerebr* OR Intracranial OR Neurolog* OR “Nervous system” OR Neurodegenerat* OR neuropath* OR seizure* OR stroke* OR “Spinal cord*”):ti,ab
